# Vitrectomy Alone Versus Anti-vascular Endothelial Growth Factor (Anti-VEGF) Therapy Prior to Vitrectomy for Diabetic Vitreous Hemorrhage: A Systematic Review and Meta-Analysis

**DOI:** 10.7759/cureus.89778

**Published:** 2025-08-11

**Authors:** Shaikha S Alhaj, Noora AlQattan, Fatma A Ismail, Fatma Alshaikh, Hassan Al Hasid

**Affiliations:** 1 Graduate Medical Education, Dubai Health, Dubai, ARE; 2 Ophthalmology, Sheikh Khalifa Medical City, Abu Dhabi, ARE; 3 Ophthalmology, Dubai Hospital, Dubai, ARE

**Keywords:** anti-vegf, diabetes, meta-analysis, vitrectomy, vitreous hemorrhage

## Abstract

Proliferative diabetic retinopathy (PDR) affects approximately 1.5% of adults with diabetes and is a leading cause of blindness due to complications such as vitreous hemorrhage. Pan-retinal photocoagulation (PRP) has been the mainstay of treatment for many years. Still, the advent of anti-vascular endothelial growth factor (anti-VEGF) agents, including ranibizumab, bevacizumab, and aflibercept, presents successful therapeutic options. This study aims to conduct a systematic review and meta-analysis to compare the efficacy of anti-VEGF therapy combined with vitrectomy versus vitrectomy alone in patients with vitreous hemorrhage due to diabetic retinopathy. Many electronic sources, such as PubMed and Google Scholar, were searched for pertinent literature. PICOS (population, intervention, comparison, outcomes, and study) criteria were applied to select the studies systematically. The Preferred Reporting Items for Systematic Reviews and Meta-Analysis (PRISMA) framework was employed to synthesize and report data. The analysis included six studies with a total of 468 patients. Results indicated a significant improvement in best-corrected visual acuity (BCVA) at three months in the anti-VEGF group compared to vitrectomy alone (MD = -0.19; p = 0.02), while no significant differences were found at one month (MD = 0.08; p = 0.50) and six months (MD = 0.03; p = 0.40). Additionally, no significant differences in recurrent vitreous hemorrhage rates were observed between groups. In conclusion, anti-VEGF drugs combined with vitrectomy showed significant improvements in BCVA at three months as compared to vitrectomy alone, indicating their potential effectiveness for diabetic vitreous hemorrhages. However, the scarcity of available research emphasizes the necessity of carrying out extensive clinical trials to validate the safety and efficacy of anti-VEGF treatment as compared to alternative treatment options.

## Introduction and background

Approximately 1.5% of adults with diabetes are diagnosed with proliferative diabetic retinopathy (PDR) [1]. Diabetic retinopathy is a complication of diabetes in which high blood sugar levels damage the small blood vessels in the retina, the light-sensitive tissue at the back of the eye, potentially leading to vision loss [2]. Diabetic retinopathy continues to be one of the primary causes of blindness among working-age adults globally. It remains one of the leading causes of blindness in working-age adults worldwide [2]. In advanced stages, such as PDR, new abnormal blood vessels form on the surface of the retina, a process known as neovascularization. These fragile vessels can rupture, leading to bleeding into the vitreous cavity, a condition known as vitreous hemorrhage [3]. Vitreous hemorrhage is prevalent among individuals diagnosed with PDR and has the potential to result in significant vision impairment. PDR is characterized by retinal neovascularization, which manifests as fibrovascular proliferation at the vitreoretinal interface, leading to vitreous hemorrhage and tractional retinal detachment [4].

Since the 1980s, pan-retinal photocoagulation (PRP) has served as the cornerstone treatment for mitigating vision loss in both proliferative diabetic retinopathy and diabetic macular edema [2]. PRP involves using laser burns to reduce abnormal blood vessel growth by targeting areas of the peripheral retina. While vitreous hemorrhage may resolve spontaneously, intervention can facilitate a more rapid vision recovery [3]. Nevertheless, PRP has potential complications during treatment, including pain, loss of peripheral vision, nyctalopia, uveal effusions, exacerbation of macular edema, vitreous hemorrhage, challenges in managing eyes with vitreous hemorrhage, and the presence of advanced cataracts [1]. However, the emergence of anti-vascular endothelial growth factor (anti-VEGF) agents (ranibizumab, bevacizumab, and aflibercept) has changed the landscape of vitreous hemorrhage in PDR treatment [5]. Anti-VEGF injections work by blocking the molecule responsible for stimulating the growth of these abnormal vessels, thereby helping reduce leakage and bleeding.

When both the clinician and the patient agree that intervention is warranted, treatment options include the administration of anti-VEGF agents to promote the regression of neovascularization during the reabsorption of the hemorrhage, or the surgical approach of vitrectomy combined with PRP to remove the vitreous hemorrhage and associated neovascular membranes [3]. Vitrectomy is a surgical procedure that removes the blood-filled vitreous gel to restore vision and allow retinal treatment.

Despite the availability of these treatments, there is uncertainty about the optimal approach to managing diabetic vitreous hemorrhage. While anti-VEGF therapy may reduce intraoperative bleeding and recurrence, vitrectomy alone is often sufficient for clearing hemorrhage. There have been several original investigations that studied the efficacy of anti-VEGF and vitrectomy versus vitrectomy alone, but there has been no systematic review and meta-analysis on this topic before. To our knowledge, no prior systematic review and meta-analysis has comprehensively compared these two strategies. Therefore, we conducted a systematic review and meta-analysis that compared the efficacy of anti-VEGF and vitrectomy versus vitrectomy alone in patients with vitreous hemorrhage due to diabetic retinopathy. Our primary outcome was best-corrected visual acuity (BCVA), a standard measure of the clearest vision a person can achieve with corrective lenses. Secondary outcomes included the recurrence of vitreous hemorrhage.

## Review

Methods

This systematic review and meta-analysis were conducted according to the guidelines provided by the Cochrane Handbook for Systematic Reviews of Interventions and Preferred Reporting Items for Systematic Reviews and Meta-Analysis (PRISMA) [6]. Ethical approval was not required, as the study utilized already published, publicly available data.

Literature Search and Screening

A literature search was conducted on PubMed and Google Scholar to find studies that compared the clinical efficacy of anti-VEGF injections plus vitrectomy versus vitrectomy alone in patients with diabetic vitreous hemorrhage. Detailed search strategies for each database are demonstrated in Table 1.

**Table 1:** Detailed search strategy of each database

Database	Search String
PubMed 251 Results	("vitreous haemorrhage"[All Fields] OR "vitreous hemorrhage"[MeSH Terms] OR ("vitreous"[All Fields] AND "hemorrhage"[All Fields]) OR "vitreous hemorrhage"[All Fields] OR (("diabete"[All Fields] OR "diabetes mellitus"[MeSH Terms] OR ("diabetes"[All Fields] AND "mellitus"[All Fields]) OR "diabetes mellitus"[All Fields] OR "diabetes"[All Fields] OR "diabetes insipidus"[MeSH Terms] OR ("diabetes"[All Fields] AND "insipidus"[All Fields]) OR "diabetes insipidus"[All Fields] OR "diabetic"[All Fields] OR "diabetics"[All Fields] OR "diabets"[All Fields]) AND ("vitreous haemorrhage"[All Fields] OR "vitreous hemorrhage"[MeSH Terms] OR ("vitreous"[All Fields] AND "hemorrhage"[All Fields]) OR "vitreous hemorrhage"[All Fields])) OR (("proliferative"[All Fields] OR "proliferatively"[All Fields] OR "proliferatives"[All Fields]) AND ("diabetic retinopathy"[MeSH Terms] OR ("diabetic"[All Fields] AND "retinopathy"[All Fields]) OR "diabetic retinopathy"[All Fields]))) AND ("vitrectomy"[MeSH Terms] OR "vitrectomy"[All Fields] OR "vitrectomies"[All Fields]) AND ("aflibercept"[Supplementary Concept] OR "aflibercept"[All Fields] OR ("bevacizumab"[MeSH Terms] OR "bevacizumab"[All Fields] OR "bevacizumab s"[All Fields]) OR ("ranibizumab"[MeSH Terms] OR "ranibizumab"[All Fields])) AND ("effect"[All Fields] OR "effecting"[All Fields] OR "effective"[All Fields] OR "effectively"[All Fields] OR "effectiveness"[All Fields] OR "effectivenesses"[All Fields] OR "effectives"[All Fields] OR "effectivities"[All Fields] OR "effectivity"[All Fields] OR "effects"[All Fields] OR (("visual"[All Fields] OR "visualisation"[All Fields] OR "visualisations"[All Fields] OR "visualise"[All Fields] OR "visualised"[All Fields] OR "visualises"[All Fields] OR "visualising"[All Fields] OR "visualization"[All Fields] OR "visualizations"[All Fields] OR "visualize"[All Fields] OR "visualized"[All Fields] OR "visualizer"[All Fields] OR "visualizers"[All Fields] OR "visualizes"[All Fields] OR "visualizing"[All Fields] OR "visually"[All Fields] OR "visuals"[All Fields]) AND ("outcome"[All Fields] OR "outcomes"[All Fields])) OR ("visual acuity"[MeSH Terms] OR ("visual"[All Fields] AND "acuity"[All Fields]) OR "visual acuity"[All Fields]))
Google Scholar 494 Results	("anti vegf" OR "bevacizumab" OR "aflibercept" OR "Ranibizumab" versus "vitrectomy") AND ("diabetic vitreous hemorrhage")

Inclusion Criteria

The inclusion criteria were: Population: Patients diagnosed with diabetic vitreous hemorrhage; Intervention: Anti-VEGF injections combined with vitrectomy; Comparison: Vitrectomy alone; Outcomes: At least one of the following: BCVA (at 1, 3, or 6 months) or recurrence of vitreous hemorrhage; Study design: Randomized controlled trials and non-randomized observational studies.

Exclusion Criteria

The studies that were excluded were: studies not involving diabetic vitreous hemorrhage or not comparing the two treatment strategies; case reports, review articles, editorials, letters, conference abstracts, animal studies, and in vitro experiments.

The inclusion and exclusion criteria were applied consistently during the screening process to ensure methodological rigor and reproducibility.

Limitations of the Literature Search

Our search did not include other relevant databases, such as Embase, Web of Science, or ClinicalTrials.gov, which may have led to the omission of potentially eligible studies. Furthermore, only articles published in English were considered, which introduces a risk of language bias.

Data Extraction and Quality Assessment

Two investigators independently extracted the following data from finalized studies: first author’s surname, year of publication, study location, type of intervention, sample size, mean age, history of diabetes, intraocular pressure (IOP), outcomes, and adverse events. Any discrepancy between the two investigators was resolved by consulting a third investigator. To assess the quality of included randomized controlled trials (RCTs), we used the Cochrane Risk of Bias tool (RoB 2.0) [7]. The risk of bias was evaluated across the following domains: randomization, deviations from intended interventions, missing outcome data, measurement of outcome, and selection of reported results. Cohort studies were appraised using the risk of bias in non-randomized studies of interventions (ROBINS-I) tool, which assesses bias across seven domains: confounding, selection of participants, classification of interventions, deviations from intended interventions, missing data, measurement of outcomes, and selection of reported results [8]. The trials were scored based on high, some concerns, or low risk of bias in each domain. Traffic light plots were created using the risk-of-bias visualization (Robvis) tool [9].

Statistical Analysis

RevMan 5.4 (Cochrane Collaboration, London, UK) was used to perform statistical analysis. A random effects model was used to report the pooled BCVA at one, three, and six months. Mean differences (MDs) and risk ratios, along with their 95% CIs, were reported for the outcomes. A p-value less than 0.05 was considered statistically significant. Heterogeneity across studies was evaluated using Higgins I², with values of I² = 25%-50% considered mild, 50%-75% as moderate, and I² > 75% as severe [10]. A leave-one-out sensitivity analysis was performed to determine whether any study highly influenced pooled effect sizes.

Results

Screening Results and Study Characteristics

**Figure 1:**
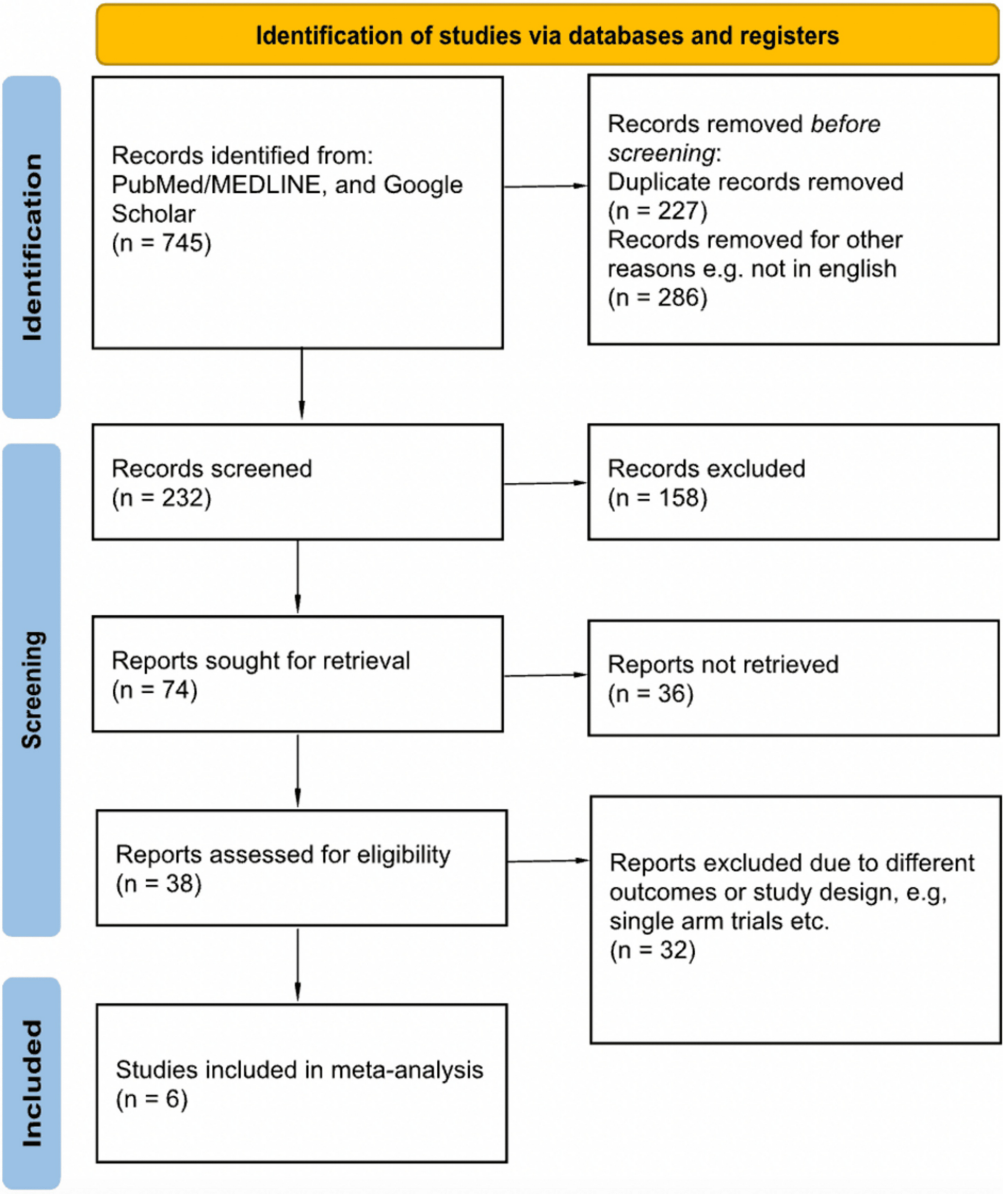
PRISMA flow diagram PRISMA: Preferred Reporting Items for Systematic Reviews and Meta-Analysis

A total of six studies were included in this systematic review and meta-analysis, including 468 patients [11-16]. The PRISMA flow diagram that summarizes the search and screening process is shown in Figure 1. The mean age of patients in studies ranged from 34.2 ± 6.7 to 58.8 ± 7.9 years. The total number of patients who received anti-VEGF injections in addition to vitrectomy was 232, while the remaining 236 patients received vitrectomy alone. The baseline characteristics of the study are demonstrated in Table 2.

**Table 2:** Characteristics of included studies * values reported as mean (SD); I = vitrectomy + anti-VEGF; C = vitrectomy alone; NR = not reported; IOP= intraocular pressure

Author Name	Year	Total Participants	Mean Age* (years)	Duration of Diabetes (years)	IOP (mmHg)*
Abd-Elhamid et al. [11]	2020	I = 17; C = 17	I = 56.4 ± 8.6; C = 58.8 ± 7.9	I = 16.4 ± 3.8; C = 15.8 ± 3.9	NR
Antoszyk et al. [12]	2020	I = 95; C = 99	I = 56 ± 12; C = 57.8 ± 11	I = 19 ± 10; C = 21 ± 11	I = 16 ± 4; C = 15 ± 3
Li et al. [13]	2022	I = 16; C = 16	I = 53.1 ± 6.3; C = 46.9 ± 11.7	NR	NR
Qu et al. [16]	2022	I = 64; C = 64	I = 52.9 ± 10.5; C = 55.3 ± 10.9	I = 8.4 ± 5.2; C = 9.0 ± 5.0	I = 14.6 ± 2.9; C = 14.9 ± 3.0
Jiang et al. [14]	2020	I = 15; C = 15	I = 55.54 ± 9.94; C = 53.5 ± 9.59	I = 13.19 ± 8.08; C = 9.88 ± 8.52	I = 12.46 ± 3.26; C = 12.3 ± 1.44
Chen et al. [15]	2020	I = 25; C = 25	I = 34.2 ± 6.7; C = 35.5 ± 5.8	I = 9.3 ± 5.2; C = 10.0 ± 5.9	NR

Risk of Bias Assessment

**Figure 2:**
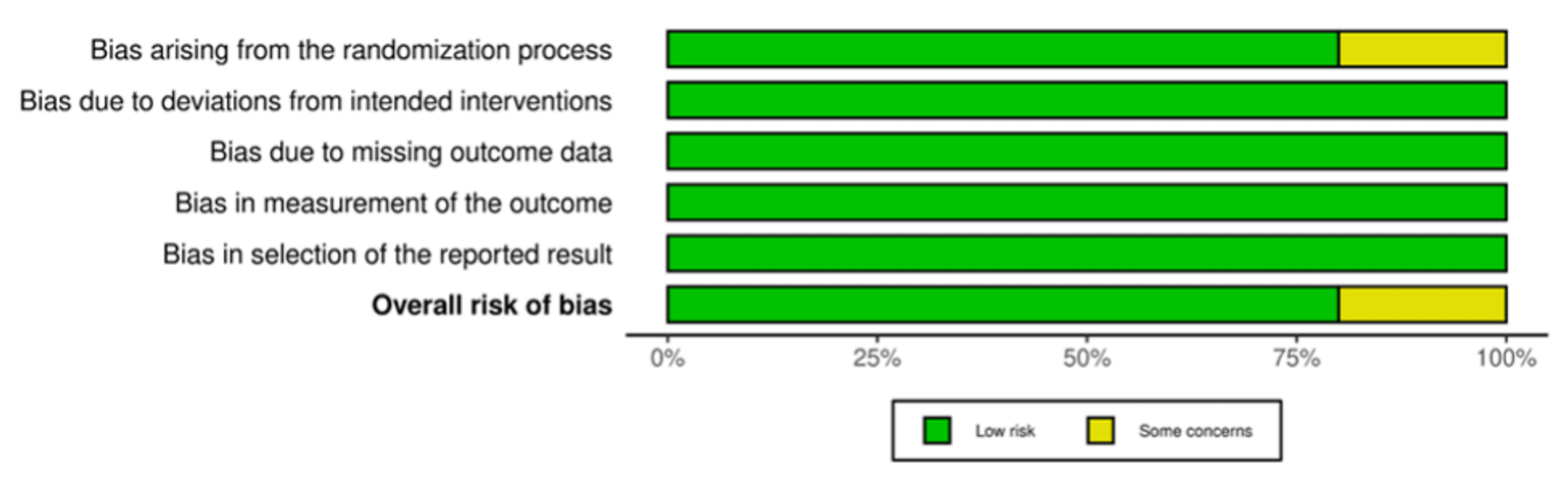
Summary light plot showing the risk of bias assessment of randomized controlled trials

**Figure 3:**
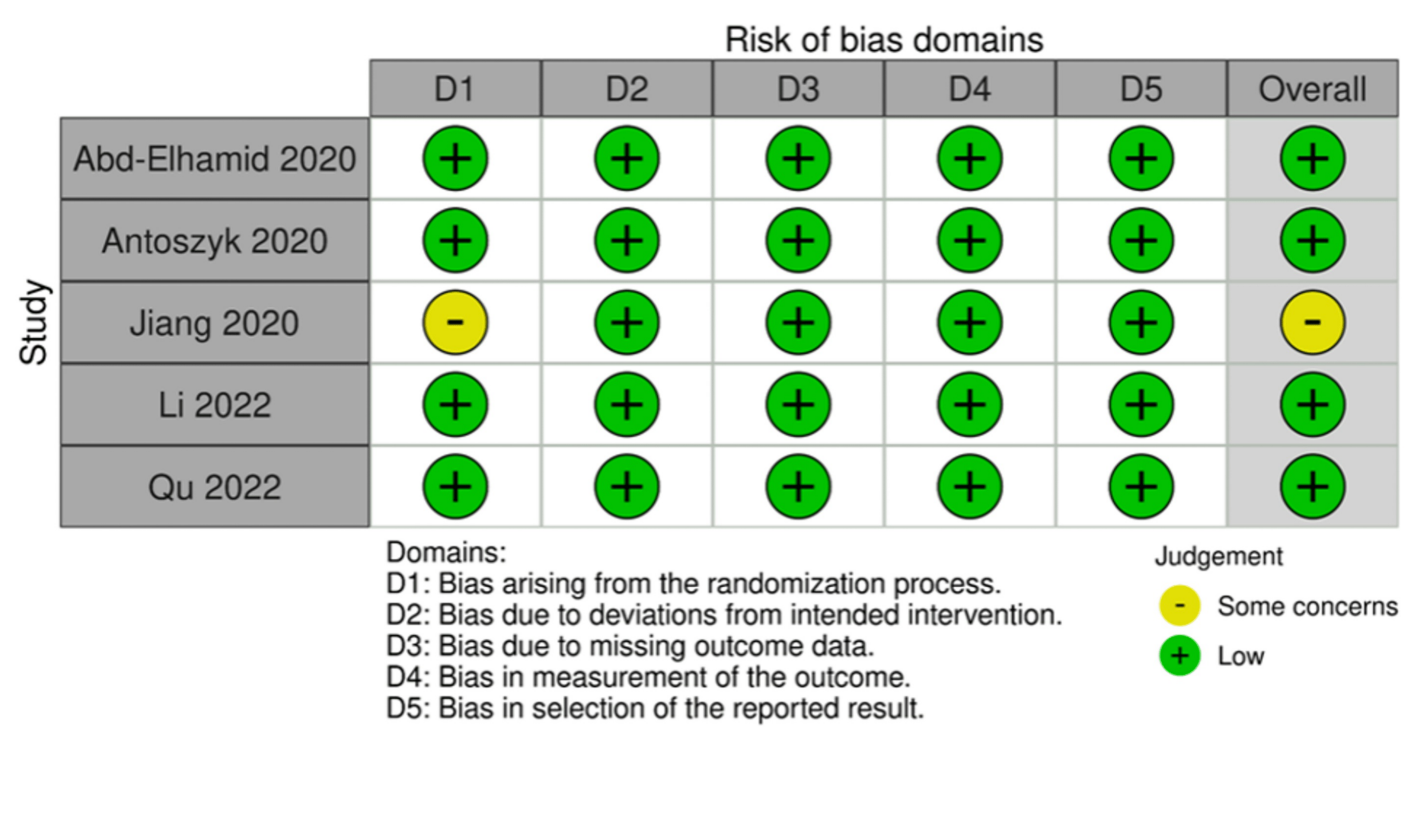
Traffic Light Plot showing the Risk of Bias Assessment of Randomized Controlled Trials Abd-Elhamid et al. [11], Antoszyk et al. [12], Jiang et al. [14], Li et al. [13], Qu et al. [16]

**Figure 4:**
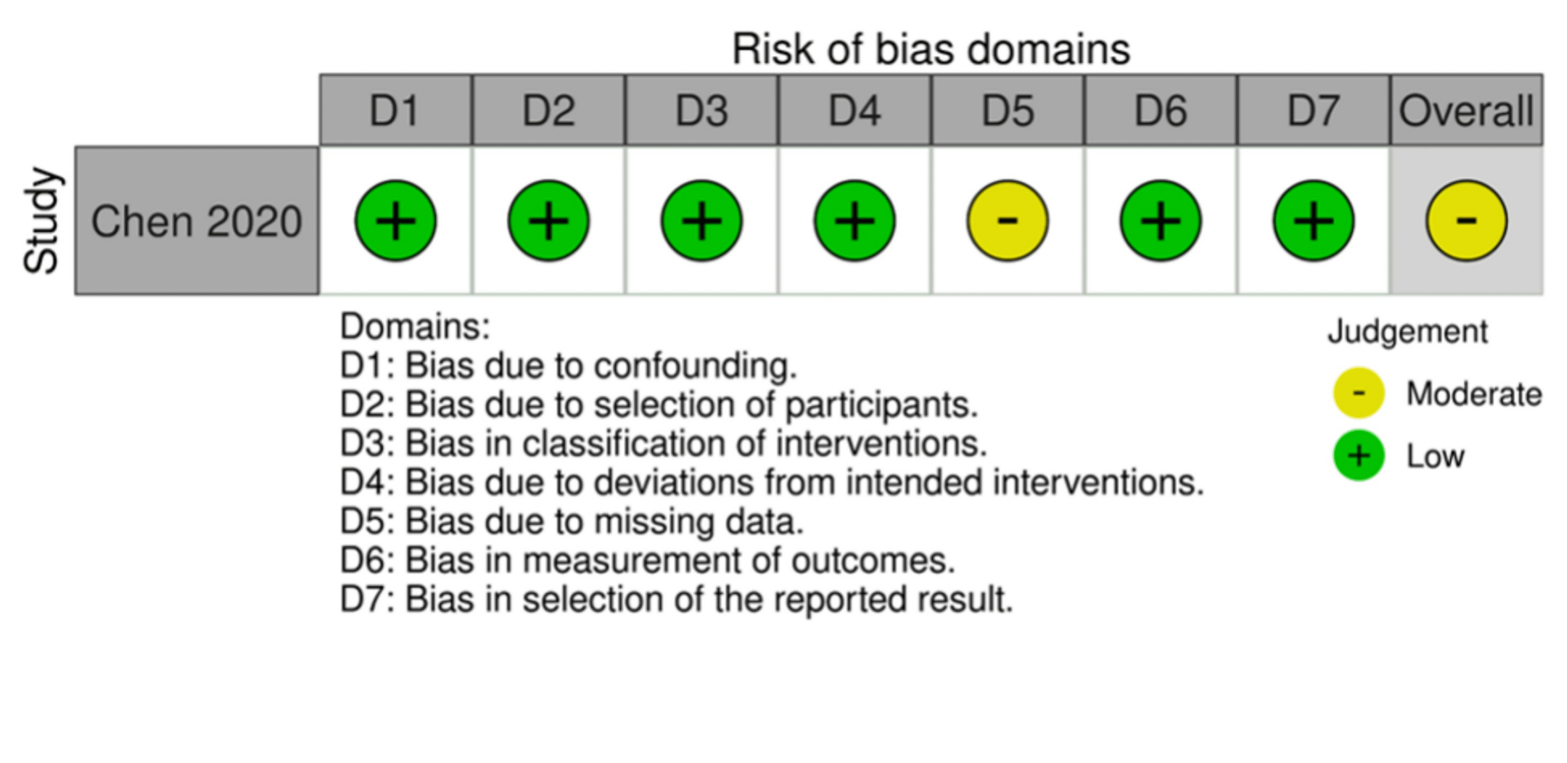
Summary light plot showing the risk of bias assessment of non-randomized studies Chen et al. [15]

**Figure 5:**
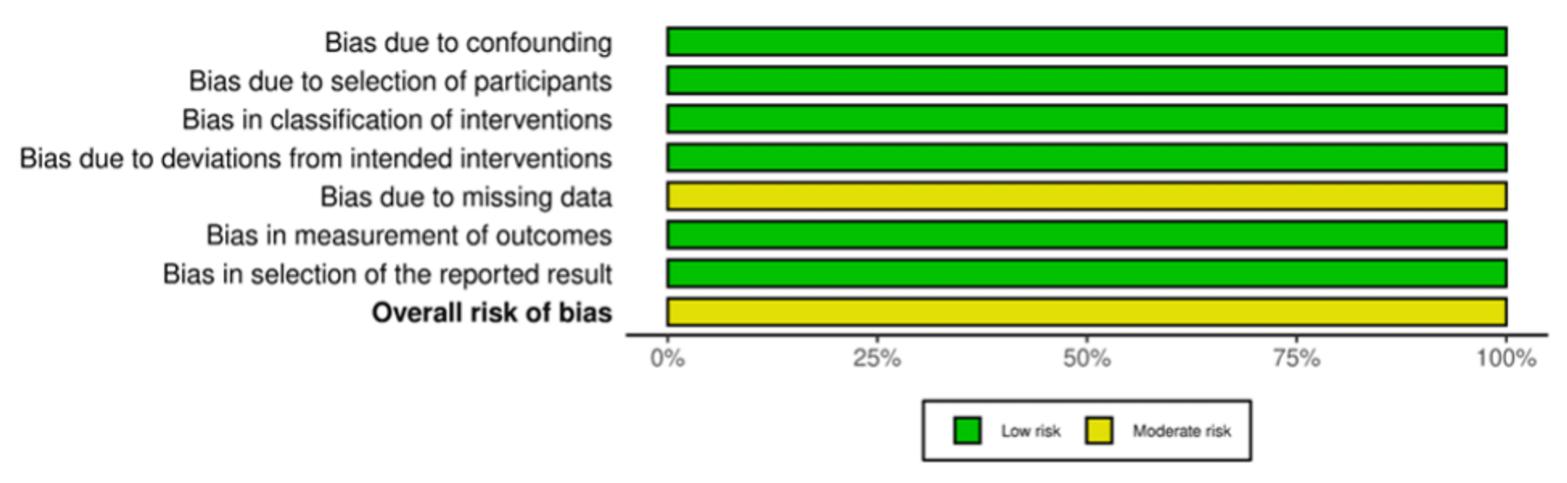
Summary light plot showing the risk of bias assessment of non-randomized studies

Five of the total included studies were RCTs. Four studies demonstrated an overall low risk of bias, while one study showed some concerns. One study was non-randomized and was evaluated using ROBINS-I. It demonstrated a moderate risk of bias. The results of the quality assessment are demonstrated in Figures 2-5.

Results of Statistical Data Synthesis

**Figure 6:**
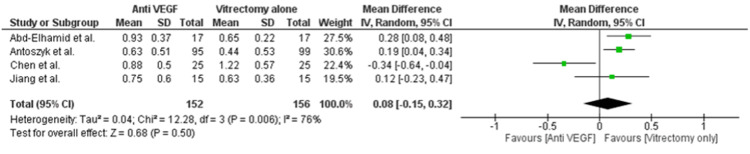
Forest plot of best corrected visual acuity at one month Abd-Elhamid et al. [11], Antoszyk et al. [12], Chen et al. [15], Jiang et al. [14]

**Figure 7:**
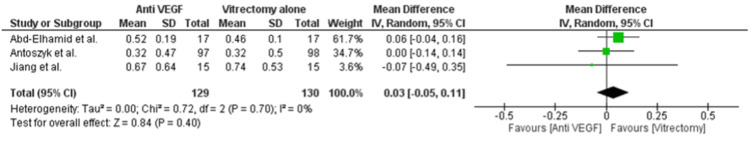
Forest plot of best corrected visual acuity at six months Abd-Elhamid et al. [11], Antoszyk et al. [12], Jiang et al. [14]

**Figure 8:**
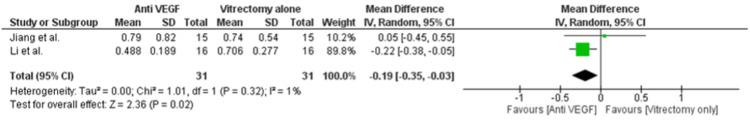
Forest plot of best corrected visual acuity at three months Jiang et al. [14], Li et al. [13]

**Figure 9:**
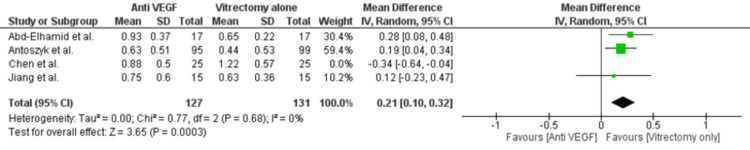
Forest plot of best corrected visual acuity at one month: leave-one-out sensitivity analysis Abd-Elhamid et al. [11], Antoszyk et al. [12], Chen et al. [15], Jiang et al. [14]

**Figure 10:**
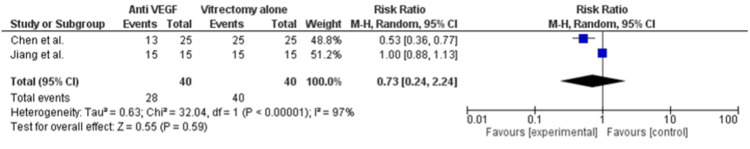
Forest Plot of recurrent vitreous hemorrhage Chen et al. [15], Jiang et al. [14]

Our pooled analysis revealed no significant treatment differences between the two groups in BCVA at 1 (MD = 0.08; 95% CI = -0.15 to 0.32; p-value = 0.50; I2 = 76%; Figure 6) and 6 months (MD = 0.03; 95% CI = -0.05 to 0.11; p-value = 0.40; I2 = 0%; Figure 7). However, significant clinical benefit in BCVA was observed at 3 months in the anti-VEGF group (MD = -0.19; 95% CI = -0.35 to -0.03; p-value = 0.02; I2 = 1%; Figure 8). Since a significant heterogeneity was observed in BCVA at one month, we conducted a leave-one-out sensitivity analysis. The heterogeneity was significantly reduced from 76% to 0% by removing Chen et al., and the results showed that anti-VEGF injections showed a significant improvement in BCVA at 1 month (MD = 0.21; 95% CI = 0.10 to 0.32; p-value = 0.0003; I2 = 0%; Figure 9). We also evaluated recurrent vitreous hemorrhages as a safety outcome reported in two studies; however, no significant treatment differences were observed (RR = 0.73; 95% CI = 0.24 to 2.24; p-value = 0.59; I2 = 97%; Figure 10). Only 1 study discussed the ocular complications, which were reported to be 2/25 in the vitrectomy alone group and 3/25 in the anti-VEGF and vitrectomy group.

Notably, substantial heterogeneity was observed in BCVA at 1 month (I² = 76%). This may stem from differences in surgical techniques, timing of anti-VEGF injections, or baseline severity of hemorrhage. Our sensitivity analysis, which excluded the study by Chen et al., reduced heterogeneity to 0%, underscoring potential methodological inconsistencies. A formal publication bias assessment, such as a funnel plot, was not feasible due to the small number of included studies (<10), limiting our ability to detect asymmetry and assess reporting bias.

Discussion

The incidence of diabetes mellitus is rapidly increasing, and this condition frequently results in substantial metabolic disease and severe complications [17]. Diabetic retinopathy is a common and specific microvascular complication of diabetes. In working-age individuals, diabetic retinopathy remains the primary cause of preventable blindness [18]. Complications from proliferative diabetic retinopathy, such as diabetic vitreous hemorrhage, are typically treated with vitrectomy, a surgical technique that removes the vitreous gel from the eye [19]. The procedure assists in clearing the hemorrhage and makes it possible for direct therapy of the underlying retinal neovascularization. Recent years have seen the emergence of anti-VEGF therapy as a vitrectomy adjuvant with the goal of suppressing VEGF to reduce neovascularization and prevent further hemorrhages [20]. VEGF is a major factor in the formation of abnormal blood vessels in the retina [21]. Anti-VEGF drugs, such as aflibercept, bevacizumab, and ranibizumab, are increasingly used in combination with vitrectomy to improve surgical outcomes and potentially enhance visual recovery [22].

In this systematic review and meta-analysis, we evaluated the efficacy of combining anti-VEGF therapy with vitrectomy compared to vitrectomy alone in patients with diabetic vitreous hemorrhage. The pooled analysis revealed no significant differences in best-corrected visual acuity (BCVA) at one and six months between the two treatment groups. Nevertheless, the anti-VEGF group showed a notable improvement in BCVA after three months. This suggests that anti-VEGF therapy may offer early benefits in improving visual outcomes, likely due to its ability to induce regression of neovascularization prior to surgery.

The efficacy of anti-VEGF medication is supported by the improvement in BCVA at three months; however, inter-study variability may be responsible for the lack of significant changes at one and six months. At one month, significant heterogeneity was reduced after excluding the study by Chen et al., revealing a notable improvement in BCVA for the anti-VEGF group. This finding suggests that differences in patient selection or surgical techniques could impact short-term outcomes, emphasizing the need for more uniform study designs.

Interestingly, no significant difference in recurrent vitreous hemorrhage was found between the two groups. This suggests that although anti-VEGF drugs enhance early visual outcomes, they may not substantially reduce the risk of postoperative complications such as recurrent bleeding. This may be attributed to the fact that anti-VEGF primarily targets neovascular regression rather than altering the structural integrity of the vitreous cavity or retinal vasculature [23].

The variable time interval between anti-VEGF administration and vitrectomy across studies may also have influenced the results. Several included studies did not report this interval clearly, and differences in timing may affect the extent of neovascular regression before surgery. Literature has shown that a longer interval can allow for more effective vascular remodeling, while shorter intervals may not yield the same benefit. Future studies should aim to standardize or stratify outcomes based on this interval to better assess its impact on visual recovery and complications.

Many important strategies are needed to improve vitreous hemorrhage outcomes. Early intervention is crucial, as prompt treatments like vitrectomy or anti-VEGF injections can prevent further vision loss by addressing neovascularization before complications arise. Combination therapy can offer a more comprehensive approach by addressing both the hemorrhage and the underlying diabetic retinopathy. One such approach is the use of anti-VEGF in conjunction with panretinal photocoagulation (PRP), which is primarily performed to reduce peripheral retinal ischemia and suppress VEGF production. One instance of this would be the use of anti-VEGF in conjunction with PRP [24]. However, PRP is not typically used to prevent macular edema; landmark studies suggest that focal or grid laser is more effective for that purpose. Rare complications of PRP exist, such as visual field constriction or macular edema, but the procedure is generally done to reduce the risk of further complications. Technological advances in surgery, particularly in vitrectomy techniques and instruments, may expedite recovery and reduce postoperative risks. Close postoperative monitoring is essential to manage any emerging issues such as intraocular pressure changes or retinal detachment [25]. Lastly, educating patients on effective diabetes management, especially strict glycemic control, is critical to preventing the progression of retinopathy and reducing the risk of future hemorrhages.

Only one of the six included studies provided detailed reporting on ocular complications [15], limiting the ability to draw firm conclusions regarding comparative safety. This paucity of safety data restricts the clinical confidence in recommending anti-VEGF therapy broadly and emphasizes the need for comprehensive safety monitoring in future trials.

The limited number of studies and divergent findings on the efficacy of anti-VEGF therapy for diabetic vitreous hemorrhage highlight the need for further research. Our understanding of the long-term consequences of combining anti-VEGF with vitrectomy remains limited by inconsistent data. To develop more precise guidelines for managing diabetic vitreous hemorrhage and optimizing patient outcomes, future research should focus on well-designed clinical trials with standardized protocols-including clear documentation of anti-VEGF timing relative to surgery.

In clinical settings, treatment decisions must consider both efficacy and accessibility. While anti-VEGF therapy may provide visual acuity benefits at three months, the added costs and procedural burden can be significant, particularly in resource-limited regions. Furthermore, there is concern regarding the potential for tractional retinal detachment (TRD), especially when anti-VEGF is administered in advanced PDR cases. The lack of data on TRD incidence in our analysis warrants caution. Therefore, clinical decisions should be individualized, balancing visual outcomes, cost, risk of TRD, and health system capacity.

## Conclusions

Anti-VEGF injections and vitrectomy demonstrated significant improvement in BVCA at one and three months compared to vitrectomy alone. Therefore, anti-VEGF injections are a potentially effective treatment for diabetic vitreous hemorrhages. However, the literature on this topic is very scarce. Future research is warranted with large-scale clinical trials to establish the efficacy and safety profile of anti-VEGF as compared to other available treatment options.
